# Striatal Dopamine Transporter Function Is Facilitated by Converging Biology of α-Synuclein and Cholesterol

**DOI:** 10.3389/fncel.2021.658244

**Published:** 2021-04-15

**Authors:** Sarah Threlfell, Amir Saeid Mohammadi, Brent J. Ryan, Natalie Connor-Robson, Nicola J. Platt, Rishi Anand, Florence Serres, Trevor Sharp, Nora Bengoa-Vergniory, Richard Wade-Martins, Andrew Ewing, Stephanie J. Cragg, Katherine R. Brimblecombe

**Affiliations:** ^1^Department of Physiology, Anatomy and Genetics, University of Oxford, Oxford, United Kingdom; ^2^Oxford Parkinson’s Disease Centre, Medical Sciences Division, University of Oxford, Oxford, United Kingdom; ^3^Department of Chemistry and Chemical Engineering, Chalmers University of Technology, Gothenburg, Sweden; ^4^University Department of Pharmacology, University of Oxford, Oxford, United Kingdom; ^5^Department of Chemistry and Molecular Biology, University of Gothenburg, Gothenburg, Sweden

**Keywords:** dopamine transporter (DAT), alpha-synuclein (SNCA), cholesteroI, striatum, Parkinson’s disease, early stage parkinsonism, dopamine uptake, galactoceramide

## Abstract

Striatal dopamine transporters (DAT) powerfully regulate dopamine signaling, and can contribute risk to degeneration in Parkinson’s disease (PD). DATs can interact with the neuronal protein α-synuclein, which is associated with the etiology and molecular pathology of idiopathic and familial PD. Here, we tested whether DAT function in governing dopamine (DA) uptake and release is modified in a human-α-synuclein-overexpressing (*SNCA*-OVX) transgenic mouse model of early PD. Using fast-scan cyclic voltammetry (FCV) in *ex vivo* acute striatal slices to detect DA release, and biochemical assays, we show that several aspects of DAT function are promoted in *SNCA*-OVX mice. Compared to background control α-synuclein-null mice (*Snca*-null), the *SNCA*-OVX mice have elevated DA uptake rates, and more pronounced effects of DAT inhibitors on evoked extracellular DA concentrations ([DA]_o_) and on short-term plasticity (STP) in DA release, indicating DATs play a greater role in limiting DA release and in driving STP. We found that DAT membrane levels and radioligand binding sites correlated with α-synuclein level. Furthermore, DAT function in *Snca*-null and *SNCA*-OVX mice could also be promoted by applying cholesterol, and using Tof-SIMS we found genotype-differences in striatal lipids, with lower striatal cholesterol in *SNCA*-OVX mice. An inhibitor of cholesterol efflux transporter ABCA1 or a cholesterol chelator in *SNCA*-OVX mice reduced the effects of DAT-inhibitors on evoked [DA]_o_. Together these data indicate that human α-synuclein in a mouse model of PD promotes striatal DAT function, in a manner supported by extracellular cholesterol, suggesting converging biology of α-synuclein and cholesterol that regulates DAT function and could impact DA function and PD pathophysiology.

## Introduction

Striatal dopamine (DA) release is regulated by mechanisms that drive activity in midbrain DA neurons in conjunction with mechanisms in striatum that act on and within DA axons (Sulzer et al., 2016). DA uptake transporters (DATs) on DA axons clear striatal DA from the extracellular space to spatially and temporally limit DA signaling (Cragg and Rice, 2004). In addition, it has become evident that striatal DATs also regulate the underlying process of DA release. Inhibition of DATs with pharmacological inhibitors not only limits the uptake but also promotes the amount of DA released (John and Jones, 2007) through a synapsin-dependent pathway (Venton et al., 2006; Kile et al., 2010) suggesting that the DAT limits vesicular mobilization for release. In addition, DATs regulate the short-term dynamic plasticity of DA release, promoting subsequent release at short inter-pulse intervals (IPIs) corresponding to high frequency firing, and limiting subsequent release at longer IPIs corresponding to low frequency firing (Condon et al., 2019).

Besides their role in regulating DA release and uptake, DATs are implicated in the etiology of Parkinson’s disease (PD). DATs offer a route of entry to DA neurons-of environmental toxins e.g., MPTP, pesticides (Lehmensiek et al., 2006; Ritz et al., 2009), and higher DAT expression and DAT: VMAT ratios promote oxidative stress *via* dopamine oxidation and consequent dopamine neuron loss (Miller et al., 1999; Masoud et al., 2015). Variations in the *DAT/SLC6A3* gene that are associated with PD susceptibility increase the expression of striatal DAT (van de Giessen et al., 2009; Richter et al., 2017). Furthermore, the DAT is electrogenic, offering depolarizing currents, which can be uncoupled from translocation of DA (Sonders et al., 1997), and so contribute to a metabolic burden, requiring ATP to re-establish ion gradients across the axonal membrane (Pissadaki and Bolam, 2013).

DAT function is regulated by post-translational modifications and lipid- and protein-binding partners (Vaughan and Foster, 2013), including α-synuclein (Lee et al., 2001) and cholesterol (Jones et al., 2012). DATs co-localize with α-synuclein in human post-mortem tissue (Bellucci et al., 2011) and many aspects of the DAT are reported to be affected by α-synuclein, including surface expression, internalization, DA translocation, and sensitivity to ligands. However, there is a lack of consensus about the impact of α-synuclein on DAT function, with conflicting evidence showing that DAT function can be enhanced (Lee et al., 2001; Chadchankar et al., 2011), decreased (Wersinger and Sidhu, 2003; Swant et al., 2011; Lundblad et al., 2012), or remains unaffected (Abeliovich et al., 2000). This lack of consensus might reflect the differing experimental preparations, assays and aspect of DAT function explored, and also might arise from the different structural forms (monomeric, oligomeric or fibrillary, soluble or aggregated) of α-synuclein that might be differently present (Alegre-Abarrategui et al., 2019) and have different pathophysiological outcomes. Determining the structural form and binding partners of α-synuclein is non-trivial and has been reviewed elsewhere (Alegre-Abarrategui et al., 2019; Alza et al., 2019). In addition, α-synuclein is in an equilibrium between cytosolic and membrane-bound states and cholesterol has been shown to affect the position of this equilibrium (Man et al., 2020). Cholesterol is a key component of highly curved membranes including synaptic vesicles, which α-synuclein can associate with (Galvagnion, 2017).

Here, we assessed whether DAT function is modified in the *SNCA*-OVX mouse model of early PD relative to *Snca*-null background control mice.* SNCA*-OVX mice are devoid of mouse α-synuclein but overexpress human wild-type α-synuclein at disease-relevant levels modeling *SNCA* locus multiplication seen in PD (Singleton et al., 2003). They show early deficits in DA release prior to DA cell loss, behavioral deficits in old age (Janezic et al., 2013), and progressive α-synuclein oligomerization with age compared to *Snca*-null mice (Bengoa-Vergniory et al., 2020) but in the absence of aggregation (Janezic et al., 2013). The control *Snca*-null mice do not differ from wild-type mice in DA release levels using our stimulation protocols (Senior et al., 2008). We assessed whether the *SNCA*-OVX PD model has altered DAT function in DA release or uptake by the DAT, and explored a potential point of convergence with cholesterol biology, using fast-scan cyclic voltammetry, molecular biology and ToF-SIMS.

## Materials and Methods

### Mice

All procedures were conducted in accordance with the United Kingdom Animals (Scientific Procedures) Act of 1986 and approved by the local ethical review panel at the Department of Physiology, Anatomy and Genetics, University of Oxford. *SNCA*-OVX mice (B6.Cg-Tg(*SNCA*)OVX37Rwm Snca^tm1Rosl^/J; Jackson Laboratories stock no. 023837) are BAC-transgenic mice that overexpress human α-synuclein from the *SNCA* genomic locus at Parkinson’s disease-relevant levels, and are back-crossed onto a mouse α-synuclein-null (*Snca*-null) background. *SNCA*-OVX and their litter-mate *Snca*-null background control mice were produced as described previously (Janezic et al., 2013). Mice were age-(3–20 months) and sex-matched throughout. Data from male and female mice were combined given there were no apparent differences between the effects of cocaine on DA release between the sexes in both genotypes (Supplementary Figure 5). DAT-Cre mice (DAT^*IRES*CRE^Jax stock number 006660) were homozygote 4-month old males, generated by crossing heterozygous mice.

### Proximity Ligation Assay (PLA)

DAT:α-synuclein PLA experiments were carried out using Duolink^®^ *in situ* kits supplied by Sigma Aldrich according to the manufacturer’s instructions, and an α-synuclein antibody (syn4D6 ab1903, Abcam), and a DAT antibody (ab5990, Abcam). Briefly, the conjugates were prepared using the DuoLink^®^ Probemaker kit by incubating 20 μl of each antibody (1 mg/ml) with the Probemaker activated oligonucleotide (Plus and Minus respectively) and conjugation buffer and leaving it overnight at room temperature. Conjugates were incubated with Probemaker stop solution for 30 min at room temperature and then suspended in Probemaker storage buffer. Paraffin-embedded tissue was prepared for fluorescent PLA^®^ by dewaxing in xylene and histoclear, rehydrating *via* graded alcohols, blocking endogenous peroxidases with 10% H_2_O_2_ for 15 min at room temperature, followed by antigen retrieval using microwave heat (10 min total) and citrate buffer pH 6 (ab93678, Abcam). All samples were incubated in Duolink^®^ block solution for 1 h at 37°C, followed by conjugates diluted in Duolink^®^ PLA diluent (both DAT and α-synuclein 1:100) overnight at 4°C. After washing in TBS + 0.05% Tween 20, samples were incubated with Duolink^®^ ligation solutions and ligase for 1 h at 37°C, before washing and incubation with Duolink^®^ amplification reagents and polymerase for 2.5 h at 37°C. Samples were then washed with Duolink^®^ wash buffer B and mounted/coverslipped with Fluorsave (Calbiochem). Images were blindly acquired at three distinct sites in dorso-medial, dorso-mid and dorso-lateral striatum from a single 5 μm coronal section per mouse, and puncta were automatically counted with ImageJ. Three mice per genotype were assessed.

### DAT Immunofluorescence

Paraffin-embedded mouse brains from *SNCA*-OVX and *Snca*-null mice were sectioned to 5 μm. Paraffin-embedded tissue was prepared for immunofluorescence by dewaxing in xylene and histoclear, rehydrating *via* graded alcohols, blocking endogenous peroxidases with 10% H_2_O_2_ for 15 min at room temperature, followed by antigen retrieval using microwave heat (10 min total) and citrate buffer pH 6 (ab93678, Abcam). The tissue was blocked for 1 h at room temp (TBS containing 1 M glycine, 10% normal goat serum and 0.1% Triton) prior to incubating in DAT primary antibody (rat anti-DAT; ab5990, Abcam) overnight at 4°C. Following washes in TBS + 0.1% Triton, the tissue was incubated in secondary antibody (Alexa Fluor 488 Goat anti-rat, Life Technologies) for 1 h at room temperature. Sections were then washed and coverslipped with Fluorsave^TM^ (Calbiochem). Images were taken blindly in dorso-mid, dorso-medial and dorso-lateral striatum from two coronal sections, three mice per genotype were quantified automatically with ImageJ.

### 3H-Cocaine Autoradiography

Mice (three per group; 3–4 months old) were sacrificed by cervical dislocation and the brains immediately collected and snap-frozen in isopentane. Coronal sections (20 μm) were sectioned on a cryostat at the level of the striatum (plates 18–24; Franklin and Paxinos, 2004), thaw-mounted onto gelatinized slides, and stored at −80°C until use. Slides were defrosted and air-dried at room temperature before being pre-incubated for 10 min in an ice-cold PBS buffer (150 mM NaCl, 10 mM NaHPO_4_, pH7.4). Slides were then incubated at 4°C for 30 min in a PBS buffer containing [^3^H]-cocaine (1 μM; Perkin Elmer). Non-specific binding was determined in presence of GBR 12935 (1 μM). After incubation, slides were washed twice in ice-cold PBS and left to dry. Slides were then put in contact with film (Kodak MR) for 6 weeks. Densitometric analysis of the autoradiograms was performed against 3H-microscales (Amersham, UK) using a computerized imaging system (MCID Core, version 7.0, Interfocus Imaging Limited, UK) and subsequently normalized to *Snca*-null.

### Western Blots

Protein extraction and Western analysis was performed as previously described (Janezic et al., 2013). Striatal tissue was homogenized in PBS (pH 7.4) containing 1% Igepal CA-630, 0.1% SDS, 0.5% sodium deoxycholate, and protease inhibitor mixture using a Tissue Tearor (Biospec Products, Inc.). Protein content was quantified using a BCA assay kit (Sigma) and proteins were analyzed by Western blotting under reducing, non-denaturing conditions. Primary antibodies used were: mouse anti-alpha synuclein 1:500 (Abcam ab1903) and rat anti-DAT 1:1,000 (Millipore; mab369) and anti-beta actin (HRP-conjugated) antibody (Abcam ab49900) at 1:50,000. Bands were visualized using horseradish peroxidase-conjugated goat anti-mouse or goat anti-rat IgG (Bio-Rad) and the chemiluminescent ECL+ kit (GE Healthcare) or immobilon ECL (Millipore). Bands were quantified using ImageJ software.

### Fast-Scan Cyclic Voltammetry

Sex- and age-matched adult mice (3–20 months) were killed *via* cervical dislocation and the brains were removed quickly on ice and transferred into ice-cold oxygenated HEPES-based buffer) in mM: 120 NaCl, 20 NaHCO_3_, 6.7 HEPES acid, 5KCL, 3.3 HEPES salt, 2 CaCl_2_, 2 MgSO_4_, 1.2 K_2_PO_4_ and 10 glucose) saturated with 95%O_2_/5% CO_2_. Three-hundred micrometer coronal slices containing striatum (+1.1 to +1.4 mm anterior of Bregma; Franklin and Paxinos, 2004) were taken and left to recover at room temperature in HEPES-based bugger for at least 1 h prior to transferring to recording chamber and to aCSF (in mM: 124 NaCl, 62 NaHCo_3_, 3.8 KCl, 2.4 CaCl_2_, 1.3 MgSO_4_, 1.3 KH_2_PO_4_ and 10 glucose) saturated with 95% O_2_/5% CO_2_ for recording. Slices were warmed to 32°C and carbon fiber microelectrode (CFM) inserted into non recording site for charging for 30 min prior to recording.

Carbon-fiber microelectrodes were manufactured in-house using borosilicate glass (GC200F-10, Harvard Apparatus) and epoxy-free carbon fiber (7 μm diameter; Goodfellow). Voltage waveform (−0.7 V to +1.3 V) was scanned at 8 Hz at 800 V/s across the recording CFM and switched out of circuit between scans using a Millar Voltammeter as described previously (Threlfell et al., 2010, 2012).

Single recording sites in either dorsomedial or dorsolateral caudate putamen (CPu) were selected and multiple stimulation paradigms were delivered in a pseudo-random order with 2.5 min intervals between stimulations. Following a period of recording to establish a stable baseline in control or drug conditions, at least three repeats of each stimuli were made within a given condition—e.g., aCSF control or drug. These repeats were then averaged within an experiment and normalized and averaged across different animals to obtain a mean effect of a drug. At least three animals per experiment were used (*N*).

To assess dopamine reuptake kinetics in *SNCA*-OVX and *Snca*-null we used two different approaches. We used exponential decay curve fits and Michaelis-Menten models to fit the falling phases of evoked DA transients using GraphPad Prism 6.0. Exponential curve fits were applied to individual transients concentration-matched between genotypes, and fitted over 1 s to extract rate constants, *k*. Conditions designed to maximize [DA]_o_ and thereby approach levels that might approach *V*_max_ were train stimuli of 20 pulses at 100 Hz delivered in the presence of K_v_^+^-channel blocker 4-AP (100 μM), nicotinic acetylcholine receptor blocker DHβE (1 μM) and D2 receptor antagonist L-741,626 (1 μM).

In experiments applying cholesterol, slices were pre-incubated for 1 h in water soluble (ws)-cholesterol (50 μg/ml) or vehicle (methyl-β-cyclodextrin; 1 mM) prior to transferring to the recording chamber. For probucol treated experiments, slices were incubated in probucol (5 μM) or DMSO vehicle control for 30 min then transferred to recording chamber with probucol (5 μM) present in the superfusate throughout recording. For nystatin treated experiments slices were incubated in nystatin (25 or 100 μg/ml) or vehicle control (DMSO) for 30 min and then transferred to recording chamber due to potential interactions with the CFM.

### Drugs

Dihydro-β-erythroidine (DHβE), nomifensine maleate salt, GBR 12935, L-741,626, 4-Aminopyridine and nystatin were purchased from Tocris Bioscience. Cocaine, methyl-β-cyclodextrin and ws-cholesterol were purchased from Sigma–Aldrich. Nystatin was purchased from Abcam. Drugs were dissolved in distilled water, DMSO (GBR 12935, L-741,626, probucol and nystatin) or 0.1 M HCl (nomifensine) to make stock aliquots at 1,000–10,000× final concentrations and stored at −20°C until required. Stock aliquots were diluted with oxygenated aCSF to the final concentration immediately before use. Nystatin and methyl-β-cyclodextrin and ws-cholesterol were prepared fresh prior to each use.

### Secondary Ion Mass Spectrometry (Tof-SIMS)

Frozen brain samples were sectioned at −20°C to 6 μm thickness and the slices containing both CPu and NAc were mounted on ITO glass slides. The brain tissue samples were stored in capped containers at −80°C until SIMS analysis. The sections were dehydrated in a vacuum desiccator for 30 min. ToF-SIMS analysis was performed using a J105 3D mass spectrometry imaging instrument (Ionoptika, Limited, U.K.). The instrument was equipped with a continuous 40 keV primary gas cluster ion beam (GCIB) to analyze the samples and clusters of CO_2_ gas (AGA, Sweden) with a nominal size of 6,000 [(CO_2_)_6,000_^+^] were utilized as the primary analysis ion beam. The ion dose density was kept below the static limit (1 × 10^13^ ions/cm^2^), in order to minimize surface damage. Spectra were obtained over a mass range of 100–1,000 Da and the mass resolution was approximately 5,000–8,000 in the range of intact phospholipids. In order to perform statistical analysis and comparison between *SNCA*-OVX and *Snca*-null mice, data were collected from three animals per genotype and from four slices per mouse. Data were normalized to the total ion counts obtained in the measured mass range.

### Statistical Analysis

All data are expressed as means ± SEM (unless otherwise stated) and the sample size, *n* = technical repeats and *N* = number of animals. The number of animals in each data set was ≥3. Parametric tests were used for data sets with equal variances and passed normality tests (D’Agostino and Pearson normality test). Subsequently, comparisons for differences in means were assessed using unpaired *t*-tests, or Mann–Whitney tests or one- or two-way ANOVAs with *post hoc* Sidak’s multiple comparison tests using GraphPad Prism 6.0. Michaelis–Menten fits used GraphPad Prism.

## Results

### DAT Function Is Potentiated by α-Synuclein

**Figure 1:**
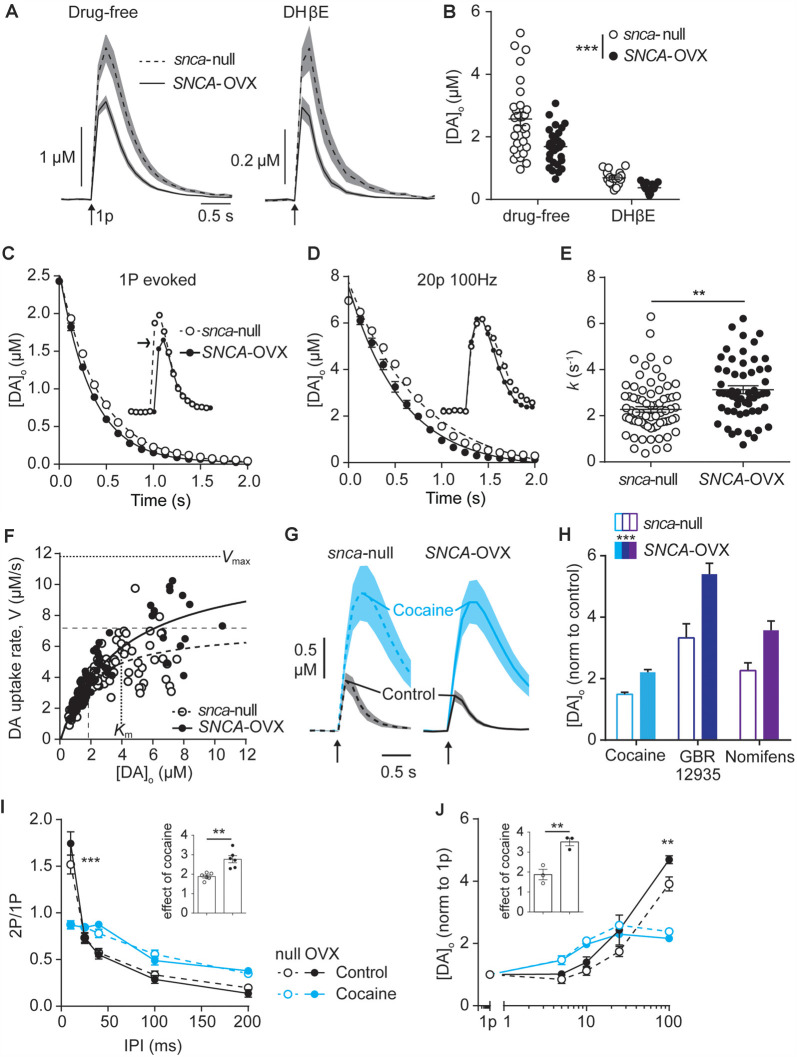
Dopamine transporters (DAT) function is potentiated by α-synuclein. **(A)** Mean [DA]_o_ ± SEM (*shaded*) evoked by single pulses (*arrow*) vs. time in caudate putamen (CPu) of *Snca*-null (dashed line) and* SNCA*-OVX (solid line) mice in drug-free conditions (*left*) or DHβE (*right*). **(B)** Summary of peak evoked [DA]_o_. ***P < 0.001, two-way ANOVA main effect of genotype: *F*_(1,90)_ = 14.54, *P* = 0.0003; drug x genotype interaction: *F*_(1,90)_ = 3.19, *P* = 0.078. **(C,D)** One-phase exponential decay curve fits for falling phases of the mean of concentration-matched [DA]_o_ transients evoked by a single pulse **(C)** or 20p 20 Hz in the presence of DHBE (1 μM), 4-aminopyridine (100 μM), L-741,626 (1 μM) **(D)**, for *Snca*-null and *SNCA*-OVX mice. *Insets*, transients offset to allow for concentration-matching *(arrow)*. Comparison of *k*, *Snca*-null vs. *SNCA*-OVX: 1p, 2.39 vs. 2.92, *F*_(1,825)_ = 64.5, *P* < 0.0001; 20p, 1.29 vs. 1.73, *F*_(1,842)_ = 53.01, *P* < 0.0001. **(E)** Summary of *k* (s^−1^) calculated for all transients, 2.34 vs. 3.13, *t*_(123)_ = 3.35, **P = 0.0011. **(F)** Maximum decay rates seen for each transient vs. [DA]_o_ at that rate for *Snca*-null (unfilled) and *SNCA*-OVX (filled). Unconstrained Michelis–Menten curve-fits for null (*dashed*) and *SNCA*-OVX (solid). *V*_max_ and *K*_m_ are indicated by horizontal and vertical lines. *V*_max_ = 7.20 vs. 11.84 μM/s, *K*_m_ = 1.82 vs. 3.97 μM, comparison of fits: *F*_(2,181)_ = 15.75, *P* < 0.001, *R*^2^ = 0.52 and 0.80. **(G)** Mean [DA]_o_ (μM) ± SEM vs. time evoked by single pulses (*arrow*) in CPu, before cocaine (control, black line) and in the presence of cocaine (blue, 5 μM). **(H)** Summary of effects of DAT inhibitors cocaine, GBR 12935 and nomifensine on 1p-evoked [DA]_o_ in *Snca*-null and *SNCA*-OVX, two-way ANOVA: effect of drug, *F*_(2,28)_ = 18.63, *P* < 0.0001; effect of genotype, *F*_(1,28)_ = 18.67, *P* = 0.0002; genotype x cocaine interaction, *F*_(2,28)_ = 0.91, *P* = 0.41. **(I)** Paired-pulse ratios (PPR) for [DA]_o_ vs. inter-pulse interval (IPI) in control conditions (DHβE; *black*), and with cocaine (*blue*) in *Snca*-null (unfilled) and *SNCA*-OVX (filled). Two-way ANOVA: condition x IPI interaction, *F*_(12,100)_ = 137.9, *P* < 0.0001, Sidak’s post-test ***P < 0.001. **(J)** Peak [DA]_o_ (normalized to condition 1p) ± SEM vs. frequency for 4p trains (5–100 Hz) in control (in the presence of DHβE; *black*) and cocaine (*blue*) in *Snca*-null (unfilled) and* SNCA*-OVX (filled). Two-way ANOVA: condition x frequency interaction, *F*_(12,40)_ = 19.56, *P* < 0.0001, Sidak’s posttest: ***P < 0.001. Insets, normalized effect of cocaine on 1p-evoked release for these datasets.

In mice expressing human α-synuclein on a mouse α-synuclein-null background (*SNCA*-OVX; Janezic et al., 2013), we used fast-scan cyclic voltammetry to test whether α-synuclein expression affected properties of DA release in *ex vivo* slices that are regulated by DAT. Initially, we ensured that we could replicate previously published finding that striatal DA release is impaired in *SNCA*-OVX compared to *Snca*-null mice by 3 months—(Janezic et al., 2013). In control conditions, [DA]_o_ release evoked by a single local electrical pulse in the dorsal striatum was 34% lower in* SNCA*-OVX compared to *Snca-*null (Figures 1A,B, 1.69 ± 0.11 μM vs. 2.57 ± 0.20 μM), in line with previous findings. Local electrical stimuli evoke DA release by direct depolarization of DA axons and indirectly by activating nAChRs on DA axons following evoked ACh release from cholinergic interneurons (Cachope et al., 2012; Threlfell et al., 2012; Wang et al., 2014). Therefore, we also tested whether the DA deficit in *SNCA*-OVX mice is present when the ACh-driven portion of DA release is prevented. In the presence of nAChR antagonism (DHβE 1 μM), levels of [DA]_o_ evoked by a single electrical pulse were lower than in drug-free media as previously published (Rice and Cragg, 2004), and, remained 34% lower in *SNCA*-OVX vs. *Snca*-null mice (Figures 1A,B; 0.37 ± 0.03 μM vs. 0.69 ± 0.05 μM).

Next, we explored DA uptake kinetics using several approaches. We first extracted decay constants, *k*, from single-phase exponential decay curves fitted to concentration-matched [DA]_o_ transients evoked by either single pulse or stimulation protocols that drive elevated [DA]_o_ (20p 100 Hz in DHβE, 4AP, L741–626). *k* was significantly greater in *SNCA*-OVX than *Snca-*null mice indicating faster uptake kinetics, for both stimulus protocols when analyzed either separately (Figure 1C) or in combination (Figures 1D,E). Second, we constructed a Michaelis–Menten-like plot, of the maximum decay rate (*V*) found on falling transients plotted vs. [DA]_o_ and found that both *V*_max_ and *K*_m_ of the best-fit curves were greater in *SNCA*-OVX than *Snca*-null mice (Figure 1F, *V*_max_: 11.8 vs. 7.2 μM/s; *K*_m_: 4.0 vs. 1.8 μM). We then tested whether the altered DAT function in *SNCA*-OVX was reflected by differential effects of DAT inhibition on [DA]_o_. Indeed, inhibition of DAT with either cocaine (5 μM), GBR 12935 (10 μM) or nomifensine (10 μM) resulted in a greater enhancement of evoked [DA]_o_ in* SNCA*-OVX vs. *Snca*-null mice (Figures 1G,H). We explored whether the changes to DAT function might be an adaptation to, or conversely arise independently from, a deficit in evoked DA release levels in *SNCA*-OVX mice (Janezic et al., 2013) as chronic DA levels can impact DAT function (Calipari et al., 2013; Siciliano et al., 2018; Brodnik et al., 2020). In particular we tested whether the effect of cocaine was greater in *SNCA*-OVX vs. *Snca*-null mice in the NAc where, in contrast to dorsal striatum, there is no deficit in evoked [DA]_o_ (Janezic et al., 2013), and found that the effect of cocaine on 1p-evoked [DA]_o_ was similarly enhanced in NAc of *SNCA*-OVX mice (Supplementary Figure 1) indicating that altered DAT function in *SNCA*-OVX mice occurs is not due to a DA release deficit.

In addition, we have recently shown that the DAT is a powerful regulator of short-term plasticity in DA release, whereby the DAT promotes a strong inverse relationship between [DA]_o_ released at a subsequently paired pulse (paired-pulse ratio) and interpulse interval (IPI; Condon et al., 2019). We tested whether elevated DAT function in *SNCA*-OVX resulted in this relationship being promoted. Indeed, we found a slightly steeper inverse relationship between paired-pulse ratio and IPI in *SNCA*-OVX vs. *Snca*-null (Figure 1I; in the presence of nicotinic receptor antagonism to prevent the confounding effects of ACh (Rice and Cragg, 2004). In turn, for short bursts of 4 pulses, there was a steeper relationship between frequency and evoked [DA]_o_ in *SNCA*-OVX vs. *Snca-null* (Figure 1J). These differences between genotypes were ameliorated by cocaine (Figures 1I,J), which weakened the relationship of [DA]_o_ to IPI and frequency in both genotypes, as seen previously in wild-type mice (Condon et al., 2019).

### DAT Availability Is Increased by α-Synuclein

**Figure 2:**
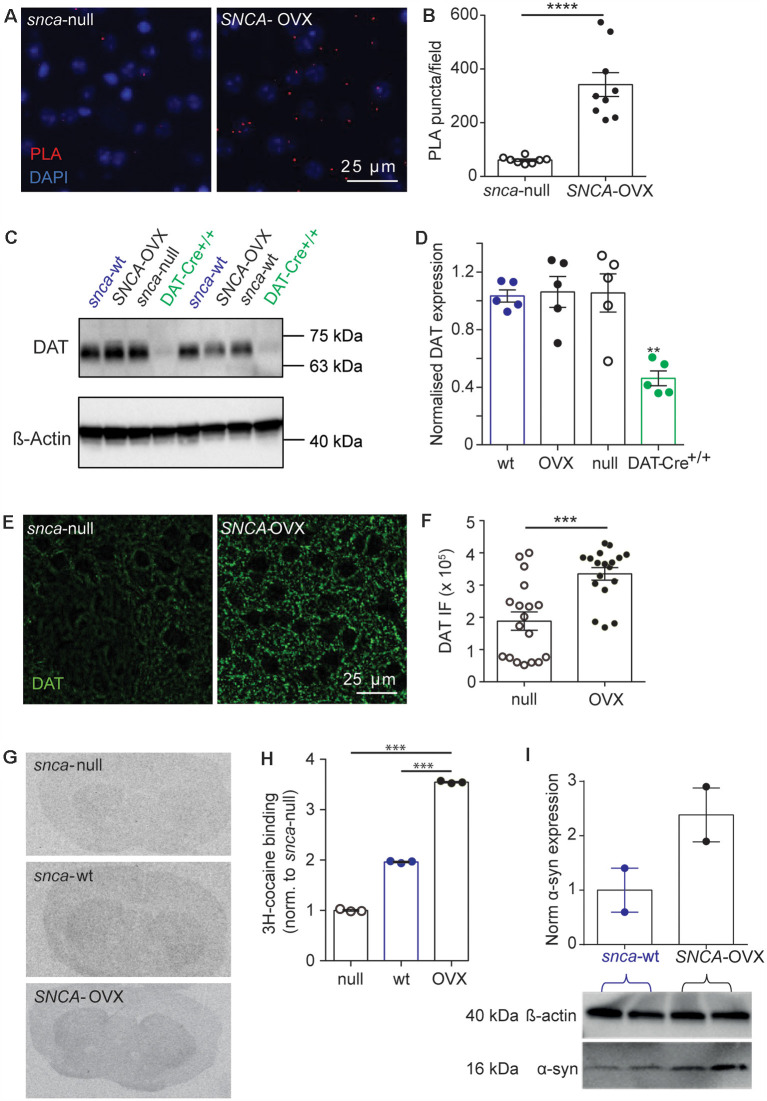
DAT availability is promoted by α-synuclein. **(A)** Representative images of PLA puncta (DAT: syn-PLA, *red*) and DAPI (*blue)* in dorsal striatum of *Snca*-null and* SNCA*-OVX mice. Scale bar, 25 μm. **(B)** Quantification of density of DAT: α-synuclein PLA puncta. Unpaired *t*-test with Welch’s correction for unequal variances, *t*_(8.156)_ = 6.285, *n* = 9 samples, *N* = 3 animals, ****P < 0.0001. **(C)** Example Western blots from striatal homogenates showing glycosylated DAT band ( 70 kDa), and actin band ( 42 kDa). **(D)** DAT levels (relative to actin) in *Snca*-wt, *Snca*-null, *SNCA*-OVX and DAT-Cre^+/+^ mice. One-way ANOVA: *F*_(3,16)_ = 1.03, *P* = 0.001. *Post hoc* Dunnett’s multiple comparisons test, C57BL/6J vs. DAT-Cre+/+, **P < 0.01. **(E)** Representative images of DAT immunofluorescence (IF) in dorsal striatum. Scale bar, 25 μm. **(F)** Quantification of DAT IF. Unpaired *t*-test, *t*_(34)_ = 4.29, ***P = 0.0001, *n* = 18, *N* = 3. **(G)** Representative autoradiograms demonstrating the distribution of ^3^H-cocaine binding sites. **(H)** Mean ± SEM ^3^H-cocaine binding sites in striatum. One-way ANOVA: *F*_(3,7)_ = 2.47, *post hoc* Dunnett’s comparisons tests for genotype, *N* = 3 per genotype, ***P < 0.001. **(I)** Western blot from striatal homogenates showing pan-species α-synuclein band (16 kDa) and actin band (42 kDa) with normalized quantification of α-synuclein expression, from *Snca*-wt and *SNCA*-OVX. *N* = 2 animals.

To understand the enhanced DAT function in* SNCA*-OVX striatum we explored how DATs and α-synuclein interact. Using a proximal ligation assay (PLA) we identified that DATs form a close spatial arrangement with α-synuclein (<40 nm) in *SNCA*-OVX mice (Figures 2A,B), supporting the hypothesis that α-synuclein and DAT associate. We tested whether α-synuclein expression increased DAT levels using western blot. However, under reducing conditions we found no difference in total striatal DAT levels between wild-type mice (*Snca*-wt), *Snca*-null, or *SNCA*-OVX mice (Figures 2C,D). To validate these data, and in particular, as a positive control for the ability of western blotting to detect differences in DAT levels, we confirmed that we could detect the lower DAT levels seen in DAT-Cre^+/+^ mice compared to controls (Bäckman et al., 2006). In parallel with these low levels of DAT we confirmed in DAT-Cre mice that uptake rates for released DA were correspondingly low and that there were only modest effects of cocaine on peak [DA]_o_ and paired-pulse ratios detected with FCV (Supplementary Figures S2A,B). Consequently, the elevated DAT function in *SNCA*-OVX mice seems not due to different level of total DAT, suggesting that DAT function might be affected at the level of the cell surface. We tested whether α-synuclein could be revealed to modify the levels of DAT levels under more native conditions using two different assays. We immunolabeled DAT in striatum and found greater levels of DAT-immunoreactivity in *SNCA*-OVX vs. *Snca*-null striatum (Figures 2E,F). We also used autoradiography with [^3^H]-cocaine to determine the relative density of striatal DAT binding sites, and found levels to be about 2.5 times higher in *SNCA*-OVX mice compared to *Snca*-null mice, with intermediate levels seen in wild-type mice *Snca*-wt; Figures 2G,H). The relative level of transporter binding, *SNCA*-OVX > *Snca*-wt > *Snca*-null tallies with the order of relative effect size of cocaine on increasing peak 1p-evoked [DA]_o_ seen with cocaine in these genotypes in our hands, which is respectively 2.5-fold in *SNCA*-OVX > 2-fold in wild-types (Condon et al., 2019) > and 1.8-fold in *Snca*-null (Figure 1). These differences between *SNCA*-OVX and *Snca*-wt mice also correlate with the relative expression levels of pan-species α-synuclein, whereby *SNCA-*OVX expresses human α-synuclein at 2-fold the level of mouse α-synuclein seen in *Snca*-wt mice (Figure 2I), as previously published (Janezic et al., 2013). Together these data illustrate that DAT and α-synuclein form a close spatial interaction and that α-synuclein promotes DAT availability to bind its ligand.

### Cholesterol Promotes DAT Function

**Figure 3:**
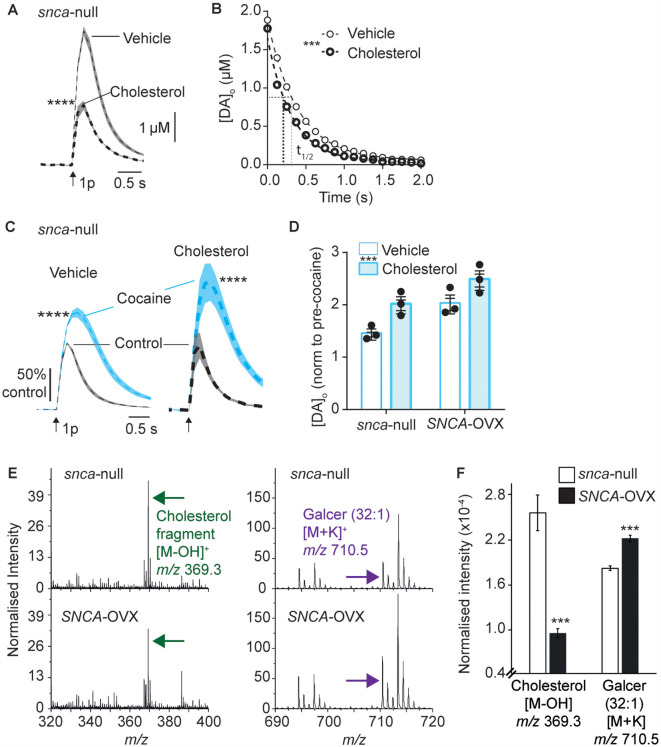
Applied cholesterol potentiates DAT function. **(A)** Mean [DA]_o_ ± SEM vs. time evoked by single pulses (*arrow*) in CPu from *Snca*-null mice incubated in vehicle or ws-cholesterol. ****P < 0.0001. **(B)** One-phase exponential decay curve fits for falling phases of mean [DA]_o_ transients concentration-matched. *k* (s^−1^) 2.54 (vehicle) vs. 3.14 (cholesterol), half-lives (*t*_(1/2)_) are indicated by for vehicle (fine line) and cholesterol (thick dotted), *F*_(1,617)_ = 46.4, ***P < 0.0001. **(C)** Mean [DA]_o_ (normalized to control conditions) ± SEM vs. time evoked by single pulses (*arrow*) in CPu of *Snca* -null mice, in control (*black*) and in the presence of cocaine (*blue*) from slices incubated in vehicle or cholesterol. ****P < 0.0001. **(D)** Summary of effects of cocaine on mean peak [DA]_o_ ± SEM, normalized to pre-cocaine condition. Two-way ANOVA: effect of cholesterol, *F*_(1,8)_ = 14.9, ***P = 0.005; effect of genotype, *F*_(1,8)_ = 15.8, *P* = 0.004; Genotype x cocaine interaction, *F*_(1,8)_ = 0.15, *P* = 0.70. **(E)** Secondary ion mass spectrometry (SIMS) analyses of *Snca* -null (top) and *SNCA*-OVX (bottom) striatum groups obtained from the striatum region of brain tissue in positive ion mode. Cholesterol (green arrow) and galactosylceramide (*galcer*; purple arrow) signal intensity (normalized to the number of selected pixels for the spectrum). **(F)** Statistical comparison of striatal lipid species cholesterol and galcer (32:1). In SNCA−/− (unfilled bars) and *SCNA*-OVX (filled bars) Mean ± SEM (normalized to the total ion count). 2-sample *T*-test, *n* = 12, ***P < 0.001.

There is a complex reciprocal relationship between α-synuclein and cholesterol, whereby cholesterol can influence α-synuclein levels and structural forms, and cholesterol levels have been reported to depend on α-synuclein expression (Barceló-Coblijn et al., 2006; Don et al., 2014; Ronzitti et al., 2014; Mazzulli et al., 2016; Galvagnion, 2017; Hsiao et al., 2017; Alecu and Bennett, 2019) and furthermore, somewhat analogously to our findings with α-synuclein and DAT function, cholesterol has been shown to promote ligand binding to DAT (Hong and Amara, 2010; Morissette et al., 2018) and enhance DA transport (Jones et al., 2012; Morissette et al., 2018). We therefore explored whether there is converging biology between α-synuclein and cholesterol and DAT function in *SNCA*-OVX mice. Firstly, we tested whether applied cholesterol could phenocopy the effect of α-synuclein level on DAT function. Application of water-soluble cholesterol (50 μg/ml) decreased [DA]_o_ evoked by a single pulse in *Snca*-null mice (Figure 3A), and increased the rate constant *k* for exponential curve-fits to the falling phase of concentration-matched transients, indicating that applied cholesterol increases DA clearance (Figure 3B, *k*, 2.54 vs. 3.14 s^−1^, *P* < 0.0001). Furthermore, applied cholesterol enhanced the effect of cocaine on peak [DA]_o_ evoked by a single pulse in *Snca*-null mice (Figure 3C) and also in *SNCA*-OVX mice (Figure 3D). There was no difference in the effect of cocaine between vehicle and drug free conditions (Supplementary Figure 3). To explore whether cholesterol levels might be modified in *SNCA*-OVX mice, we measured cholesterol content of striatal tissue membranes using ToF-SIMS. We found that levels of cholesterol in *SNCA*-OVX mice were significantly lower than those in *Snca*-null mice, whereas levels of galactosylceremide (32:1) were higher (Figures 3E,F), which was not due to any difference in glucocerebrosidase (GBA) activity, unlike previous findings (Galvagnion, 2017; Supplementary Figure 4).

**Figure 4:**
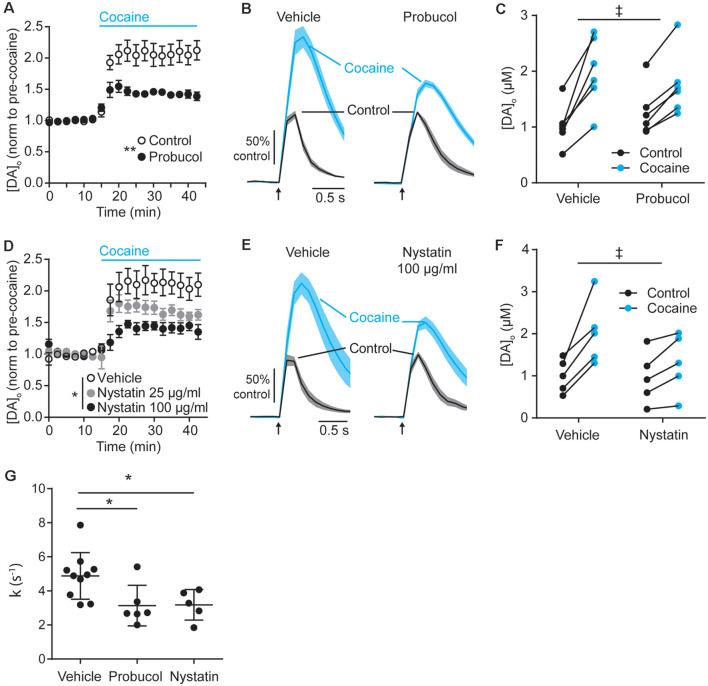
Decreasing extracellular cholesterol in *SNCA*-OVX striatum decreases DAT control of DA release. **(A–D)** Mean peak [DA]_o_ ± SEM evoked by 1p vs. time (min) in CPu of *SNCA*-OVX mice incubated in vehicle control (unfilled circles) or **(A)** Probucol (black), **(D)** Nystatin 25 μg/ml (gray), or 100 μg/ml (black). Cocaine (5 μM) added at 12.5 min (blue bar). Two-way ANOVAs: **(A)** repeated measures for time, time x probucol interaction, *F*_(17,170)_ = 10.13, *P* < 0.0001; effect of probucol, *F*_(1,10)_ = 14.19, **P = 0.0037. **(D)** time x nystatin interaction, *F*_(34,204)_ = 4.95, *P* < 0.0001; effect of nystatin, *F*_(2,12)_ = 5.98, *P = 0.016. **(B–E)** Mean [DA]_o_ (normalized to control conditions) ± SEM (shaded) vs. time evoked by single pulses (*arrow*) in CPu of *SNCA*-OVX mice from slices incubated in vehicle or **(C)** probucol (5 μM) or **(E)** nystatin 100 μg/ml (thick line) in control condition (black) or with cocaine (blue). **(C–F)** [DA]_o_ before and after cocaine from slices treated with vehicle and **(C)** probucol or **(F)** nystatin 100 μg/ml. Two-way ANOVA, repeated measures by cocaine treatment: **(C)** probucol x cocaine interaction, *F*_(1,10)_ = 6.51, ^‡^*P* = 0.029; **(F)** nystatin x cocaine interaction, *F*_(1,8)_ = 7.16, ^‡^*P* = 0.028. **(G)** Summary of *k* (s^−1^) for averaged falling phase transients in vehicle, probucol, or nystatin. One-way ANOVA: *F*_(2,18)_ = 5.17, *P* = 0.017, Dunnett’s post-tests: vehicle vs. probucol, *P = 0.024; vehicle vs. nystatin, *P = 0.038.

The finding that cholesterol content is lower in* SNCA*-OVX mice might at first seem counterintuitive, given that DAT function is enhanced in *SNCA*-OVX mice and by application of ws-cholesterol. However, these findings would be consistent with α-synuclein (soluble monomeric and oligomeric forms) being able to potentiate cholesterol efflux, *via* the ATP-binding cassette (ABC) transporter ABCA1 (Hsiao et al., 2017). Elevated cholesterol efflux could reduce cellular cholesterol level, consistent with the low cellular cholesterol content seen in *SNCA*-OVX measured by ToF-SIMS, and cholesterol efflux could promote extracellular cholesterol, consistent with elevated DAT function seen in *SNCA*-OVX mice. To test whether elevated DAT function in *SNCA*-OVX mice is due to enhanced extracellular cholesterol we tested whether an inhibitor of ABCA1 activity (probucol, 5 μM) could decrease the effect of cocaine in peak [DA]_o_. After incubation of striatal slices from *SNCA*-OVX mice in probucol, we found that the effect of cocaine was less than in vehicle-treated slices (Figures 4A–C). In addition, we tested whether nystatin (25–100 μg/ml), which chelates extracellular/sequesters lipid raft cholesterol, could also decrease the effect of cocaine. Indeed, nystatin, decreased the effect of cocaine on peak [DA]_o_ in a concentration-dependent manner (Figures 4D–F), and both nystatin and probucol led to a significant slowing of the falling phases of DA transients (Figure 4G). Together these data support the hypothesis that α-synuclein potentiates DAT function *via* its ability to potentiate cholesterol efflux *via* the ABCA1 transporter, leading to an elevation in extracellular cholesterol that in turn potentiates DAT function.

## Discussion

Here, we report that striatal DAT function is enhanced in the *SNCA*-OVX mouse model of PD, compared to *Snca-*null background controls. Furthermore, we show changes to cholesterol and other lipid levels, that can directly impact DAT function. These findings demonstrate that the intersecting biologies of α-synuclein and cholesterol can powerfully regulate DAT function.

In *SNCA*-OVX mice, we found close spatial localization of α-synuclein and DAT, and increased DAT function in several aspects relative to *Snca*-null controls. DAT function in wild-type mice represented an intermediate level between the extremes of *SNCA*-OVX and *Snca*-null mice. Extracellular DA clearance kinetics were enhanced, the effects of DAT inhibitors on peak [DA]_o_ were promoted, and there was enhanced facilitation of DA release at short IPIs in *SNCA*-OVX mice. Correspondingly, we found evidence of elevated membrane DAT levels, from *in situ* autoradiography and DAT-immunocytochemistry, but not greater total DAT expression. We found that both the *V*_max_ and *K*_m_ for DA uptake were greater than in *Snca*-null control mice. These data therefore indicate that α-synuclein is promoting DAT availability, and also changing DA binding. These outcomes could also potentially be driven by an increase in forward trafficking of DAT to the membrane, a decrease in reverse trafficking and/or a change in conformation, including the relative outward- vs. inward-facing DAT. Our findings that *K*_m_ appears increased in *SNCA*-OVX mice in addition to *V*_max_, appears to indicate that α-synuclein not only increases the number of DA-translocation sites, but also affects how DA binds to DAT. One candidate explanation might be an underlying change to the stoichiometry of DATs e.g., an increase in monomeric/oligomeric (dimer or tetramer) ratio, which was not assessed here but is known to affect the number of actively translocating DAT units and their availability to bind substrate (Gur et al., 2017; Zhen and Reith, 2018). Interestingly, cholesterol has been shown to affect how DA binds to DAT by promoting an outward facing confirmation (Hong and Amara, 2010; Jones et al., 2012). Intriguingly, our lipid analyses identified significantly lower levels of cellular cholesterol in *SNCA*-OVX mice, which is consistent with a report that soluble monomeric α-synuclein promotes cholesterol efflux, *via* the ABCA1 transporter (Hsiao et al., 2017). Potentially, the level of ceramide and cholesterol are related in a way that an increasing level of ceramide at the cell membrane promotes the release of cholesterol to the extracellular space, resulting in a lower level of cholesterol at the cell membrane (Mohammadi et al., 2018). Cholesterol efflux *via* ABCA1 transporter can occur from neurons or astrocytes (Lee et al., 2020). Correspondingly, we found that probucol, which decreases ABCA1 transporter function, or a cholesterol chelator, decreased the effect of cocaine on elevating peak evoked [DA]_o_. These findings together suggest that the elevated DAT function in *SNCA*-OVX mice is dependent on extracellular cholesterol, reflected in reduced membrane cholesterol levels. This hypothesis should be explored in future work and in other PD models.

These findings support a converging biology of α-synuclein, cholesterol and DAT, but the precise mechanism is still to be characterized. DATs are subject to extensive post-translation modifications (phosphorylation, palmitoylation, glycosylation, ubiquitination), express many regulatory binding domains (CRAC, SH3) and associate with other molecular regulators of DA release (D2Rs, RIM, synt-1; Vaughan and Foster, 2013). Both cholesterol and α-synuclein have scope to affect many of these parameters both directly and indirectly e.g., α-synuclein and cholesterol affecting VGCC function (Ronzitti et al., 2014), which in turn interacts with DAT function (Kile et al., 2010; Cameron et al., 2015). Furthermore, although DAT expression is restricted to DA axons, cholesterol and α-synuclein are more ubiquitous. Therefore, our observed effects of α-synuclein induced cholesterol efflux might involve other interacting striatal cellular networks, neuronal and non-neuronal.

The overall up-regulation of DAT function in *SNCA*-OVX mice also offers insights into another previous observation in this mouse model. DATs can apparently limit the recruitment of vesicles for release (Venton et al., 2006; Kile et al., 2010), and we have previously shown that vesicles within DA axons in *SNCA*-OVX mice are less dispersed and more tightly clustered, and that DA releasability is restricted (Janezic et al., 2013). Elevated DAT function could be a mechanism that contributes to this redistribution of DA vesicles and limited DA release.

We note that the increases to DAT function in *SNCA*-OVX mice, compared to background control mice, also similar parallel effects of knocking down a different protein in DA neurons, the Ca^2+^ binding protein calbindin-D28k (calb1; Brimblecombe et al., 2019). Calb1 expression in midbrain DA neurons is negatively correlated with vulnerability to degeneration in PD, being lower in SN neurons that are more vulnerable. A multiple hit hypothesis has been suggested for driving DA neuron degeneration in PD, arising from hits that include α-synuclein burden, Ca^2+^ burden and oxidative stress due to DA load after uptake (Sulzer, 2007; Mosharov et al., 2009; Post et al., 2018; Surmeier, 2018). We speculate that the enhanced DA uptake rates caused by α-synuclein could have important implications for pathology in Parkinson’s. For example, increased DA uptake would decrease the extracellular availability of DA for acting on post-synaptic targets, exacerbating the effects of a deficit in DA release. Furthermore, elevated DAT function might increase intracellular levels of DA and provide an additional source of oxidative stress due to production of toxic metabolites, especially if coupled with low VMAT levels (Miller et al., 1999; Masoud et al., 2015). Our findings that both cholesterol and α-synuclein function are linked to DA uptake biology, indicate that the pathological gain of each individual hit will not act independently, but co-operatively through biological interactions.

In conclusion, we show that human α-synuclein promotes multiple facets of the ways in which DATs regulate DA release and extracellular levels, through mechanisms that depend on extracellular cholesterol. The interacting biology of α-synuclein, cholesterol and DATs leads to changes to DAT function and DA signaling in this mouse model of PD that will diminish DA output, and could potentiate the burden of cytosolic DA, potentially promoting vulnerability to degeneration.

## Data Availability Statement

The raw data supporting the conclusions of this article will be made available by the authors, without undue reservation.

## Ethics Statement

The animal study was reviewed and approved by University of Oxford DPAG/EP AWERB.

## Author Contributions

KB, ST, and SC conceived the study. ST, AM, BR, NC-R, NP, RA, FS, TS, NB-V, and KB acquired and analyzed data. RW-M, AE, and TS contributed resources and commented on the manuscript. KB and SC co-wrote the manuscript. All authors contributed to the article and approved the submitted version.

## Conflict of Interest

The authors declare that the research was conducted in the absence of any commercial or financial relationships that could be construed as a potential conflict of interest.

## References

[B1] Abeliovich A. Schmitz Y. Fariñas I. Choi-Lundberg D. Ho W. H. Castillo P. E.. (2000). Mice lacking α-synuclein display functional deficits in the nigrostriatal dopamine system. Neuron 25, 239–252. 10.1016/s0896-6273(00)80886-7 10707987

[B2] Alecu I. Bennett S. A. L. (2019). Dysregulated lipid metabolism and its role in α-synucleinopathy in Parkinson’s disease. Front. Neurosci. 13:328. 10.3389/fnins.2019.00328 31031582 PMC6470291

[B3] Alegre-Abarrategui J. Brimblecombe K. R. Roberts R. F. Velentza-Almpani E. Tilley B. S. Bengoa-Vergniory N.. (2019). Selective vulnerability in α-synucleinopathies. Acta Neuropathol. 138, 681–704. 10.1007/s00401-019-02010-2 31006067 PMC6800835

[B4] Alza N. P. Iglesias González P. A. Conde M. A. Uranga R. M. Salvador G. A. (2019). Lipids at the crossroad of α-synuclein function and dysfunction: biological and pathological implications. Front. Cell. Neurosci. 13:175. 10.3389/fncel.2019.00175 31118888 PMC6504812

[B5] Bäckman C. M. Malik N. Zhang Y. Shan L. Grinberg A. Hoffer B. J.. (2006). Characterization of a mouse strain expressing Cre recombinase from the 3’ untranslated region of the dopamine transporter locus. Genesis 44, 383–390. 10.1002/dvg.20228 16865686

[B6] Barceló-Coblijn G. Golovko M. Y. Weinhofer I. Berger J. Murphy E. J. (2006). Brain neutral lipids mass is increased in α-synuclein gene-ablated mice. J. Neurochem. 101, 132–141. 10.5435/JAAOS-D-17-00026 17250686

[B7] Bellucci A. Navarria L. Falarti E. Zaltieri M. Bono F. Collo G.. (2011). Redistribution of DAT/α-synuclein complexes visualized by “*in situ*” proximity ligation assay in transgenic mice modeling early Parkinson’s disease. PLoS One 6:e27959. 10.1371/journal.pone.0027959 22163275 PMC3233557

[B8] Bengoa-Vergniory N. Faggiani E. Ramos-Gonzalez P. Kirkiz E. Connor-Robson N. Brown L. V.. (2020). CLR01 protects dopaminergic neurons *in vitro* and in mouse models of Parkinson’s disease. Nat. Commun. 11:4885. 10.1038/s41467-020-18689-x 32985503 PMC7522721

[B9] Brimblecombe K. R. Vietti-Michelina S. Platt N. J. Kastli R. Hnieno A. Gracie C. J.. (2019). Calbindin-D28K limits dopamine release in ventral but not dorsal striatum by regulating Ca^2+^ availability and dopamine transporter function. ACS Chem. Neurosci. 10, 3419–3426. 10.1021/acschemneuro.9b00325 31361457 PMC6706870

[B10] Brodnik Z. D. Xu W. Batra A. Lewandowski S. I. Ruiz C. M. Mortensen O. V.. (2020). Chemogenetic manipulation of dopamine neurons dictates cocaine potency at distal dopamine transporters. J. Neurosci. 40, 8767–8779. 10.1523/JNEUROSCI.0894-20.2020 33046544 PMC7643298

[B11] Cachope R. Mateo Y. Mathur B. N. Irving J. Wang H.-L. Morales M.. (2012). Selective activation of cholinergic interneurons enhances accumbal phasic dopamine release: setting the tone for reward processing. Cell Rep. 2, 33–41. 10.1016/j.celrep.2012.05.011 22840394 PMC3408582

[B12] Calipari E. S. Ferris M. J. Salahpour A. Caron M. G. Jones S. R. (2013). Methylphenidate amplifies the potency and reinforcing effects of amphetamines by increasing dopamine transporter expression. Nat. Commun. 4:2720. 10.1038/ncomms3720 24193139 PMC4017736

[B13] Cameron K. N. Solis E. Jr. Ruchala I. De Felice L. J. Eltit J. M. (2015). Amphetamine activates calcium channels through dopamine transporter-mediated depolarization. Cell Calcium 58, 457–466. 10.1016/j.ceca.2015.06.013 26162812 PMC4631700

[B14] Chadchankar H. Ihalainen J. Tanila H. Yavich L. (2011). Decreased reuptake of dopamine in the dorsal striatum in the absence of α-synuclein. Brain Res. 1382, 37–44. 10.1016/j.brainres.2011.01.064 21276428

[B15] Condon M. D. Platt N. J. Zhang Y.-F. Roberts B. M. Clements M. A. Vietti-Michelina S.. (2019). Plasticity in striatal dopamine release is governed by release-independent depression and the dopamine transporter. Nat. Commun. 10:4263. 10.1038/s41467-019-12264-9 31537790 PMC6753151

[B16] Cragg S. J. Rice M. E. (2004). DAncing past the DAT at a DA synapse. Trends Neurosci. 27, 270–277. 10.1016/j.tins.2004.03.011 15111009

[B17] Don A. S. Hsiao J.-H. T. Bleasel J. M. Couttas T. A. Halliday G. M. Kim W. S. (2014). Altered lipid levels provide evidence for myelin dysfunction in multiple system atrophy. Acta Neuropathol. Commun. 2:150. 10.1186/s40478-014-0150-6 25358962 PMC4228091

[B37] Franklin K. B. J. Paxinos G. (2004). Mouse Brain in Stereotaxic Coordinates. San Diego, CA: Academic Press.

[B18] Galvagnion C. (2017). The Role of lipids interacting with α-synuclein in the pathogenesis of Parkinson’s disease. J. Parkinsons Dis. 7, 433–450. 10.3233/JPD-171103 28671142

[B19] Gur M. Cheng M. H. Zomot E. Bahar I. (2017). Effect of dimerization on the dynamics of neurotransmitter:sodium symporters. J. Phys. Chem. B 121, 3657–3666. 10.1021/acs.jpcb.6b09876 28118712 PMC5402697

[B20] Hong W. C. Amara S. G. (2010). Membrane cholesterol modulates the outward facing conformation of the dopamine transporter and alters cocaine binding. J. Biol. Chem. 285, 32616–32626. 10.1074/jbc.M110.150565 20688912 PMC2952264

[B21] Hsiao J.-H. T. Halliday G. M. Kim W. S. (2017). α-synuclein regulates neuronal cholesterol efflux. Molecules 22:1769. 10.3390/molecules22101769 29048372 PMC6151759

[B22] Janezic S. Threlfell S. Dodson P. D. P. D. Dowie M. J. M. J. Taylor T. N. Potgieter D.. (2013). Deficits in dopaminergic transmission precede neuron loss and dysfunction in a new Parkinson model. Proc. Natl. Acad. Sci. U S A 110, E4016–E4025. 10.1073/pnas.1309143110 24082145 PMC3801069

[B23] John C. E. Jones S. R. (2007). Voltammetric characterization of the effect of monoamine uptake inhibitors and releasers on dopamine and serotonin uptake in mouse caudate-putamen and substantia nigra slices. Neuropharmacology 52, 1596–1605. 10.1016/j.neuropharm.2007.03.004 17459426 PMC2041899

[B24] Jones K. T. Zhen J. Reith M. E. A. (2012). Importance of cholesterol in dopamine transporter function. J. Neurochem. 123, 700–715. 10.1111/jnc.12007 22957537 PMC3517300

[B25] Kile B. M. Guillot T. S. Venton B. J. Wetsel W. C. Augustine G. J. Wightman R. M. (2010). Synapsins differentially control dopamine and serotonin release. J. Neurosci. 30, 9762–9770. 10.1523/JNEUROSCI.2071-09.2010 20660258 PMC2923550

[B26] Lee F. J. S. Lie F. Pristupa Z. B. Niznik H. B. (2001). Direct binding and functional coupling of α-synuclein to the dopamine transporters accelerate dopamine-induced apoptosis. FASEB J. 15, 916–926. 10.1096/fj.00-0334com 11292651

[B27] Lee J. A. Hall B. Allsop J. Alqarni R. Allen S. P. (2020). Lipid metabolism in astrocytic structure and function. Semin. Cell Dev. Biol. [Epub ahead of print]. 10.1016/j.semcdb.2020.07.017 32773177

[B28] Lehmensiek V. Tan E.-M. Liebau S. Lenk T. Zettlmeisl H. Schwarz J.. (2006). Dopamine transporter-mediated cytotoxicity of 6-hydroxydopamine *in vitro* depends on expression of mutant α-synucleins related to Parkinson’s disease. Neurochem. Int. 48, 329–340. 10.1016/j.neuint.2005.11.008 16406146

[B29] Lundblad M. Decressac M. Mattsson B. Bjorklund A. (2012). Impaired neurotransmission caused by overexpression of α-synuclein in nigral dopamine neurons. Proc. Natl. Acad. Sci. U S A 109, 3213–3219. 10.1073/pnas.1200575109 22315428 PMC3295273

[B30] Man W. K. De Simone A. Barritt J. D. Vendruscolo M. Dobson C. M. Fusco G. (2020). A role of cholesterol in modulating the binding of α-synuclein to synaptic-like vesicles. Front. Neurosci. 14:18. 10.3389/fnins.2020.00018 32063829 PMC7000551

[B31] Masoud S. T. Vecchio L. M. Bergeron Y. Hossain M. M. Nguyen L. T. Bermejo M. K.. (2015). Increased expression of the dopamine transporter leads to loss of dopamine neurons, oxidative stress and l-DOPA reversible motor deficits. Neurobiol. Dis. 74, 66–75. 10.1016/j.nbd.2014.10.016 25447236 PMC4505366

[B32] Mazzulli J. R. Zunke F. Isacson O. Studer L. Krainc D. (2016). α-synuclein-induced lysosomal dysfunction occurs through disruptions in protein trafficking in human midbrain synucleinopathy models. Proc. Natl. Acad. Sci. U S A 113, 1931–1936. 10.1073/pnas.1520335113 26839413 PMC4763774

[B33] Miller G. W. Gainetdinov R. R. Levey A. I. Caron M. G. (1999). Dopamine transporters and neuronal injury. Trends Pharmacol. Sci. 20, 424–429. 10.1016/s0165-6147(99)01379-6 10498956

[B34] Mohammadi A. S. Li X. Ewing A. G. (2018). Mass spectrometry imaging suggests that cisplatin affects exocytotic release by alteration of cell membrane lipids. Anal. Chem. 90, 8509–8516. 10.1021/acs.analchem.8b01395 29912552

[B35] Morissette M. Morin N. Rouillard C. Di Paolo T. (2018). Membrane cholesterol removal and replenishment affect rat and monkey brain monoamine transporters. Neuropharmacology 133, 289–306. 10.1016/j.neuropharm.2018.01.039 29407218

[B36] Mosharov E. V. Larsen K. E. Kanter E. Phillips K. A. Wilson K. Schmitz Y.. (2009). Interplay between cytosolic dopamine, calcium and α-synuclein causes selective death of substantia nigra neurons. Neuron 62, 218–229. 10.1016/j.neuron.2009.01.033 19409267 PMC2677560

[B38] Pissadaki E. K. Bolam J. P. (2013). The energy cost of action potential propagation in dopamine neurons: clues to susceptibility in Parkinson’s disease. Front. Comput. Neurosci. 7:13. 10.3389/fncom.2013.00013 23515615 PMC3600574

[B39] Post M. R. Lieberman O. J. Mosharov E. V. (2018). Can interactions between α-synuclein, dopamine and calcium explain selective neurodegeneration in Parkinson’s disease? Front. Neurosci. 12:161. 10.3389/fnins.2018.00161 29593491 PMC5861202

[B40] Rice M. E. Cragg S. J. (2004). Nicotine amplifies reward-related dopamine signals in striatum. Nat. Neurosci. 7, 583–584. 10.1038/nn1244 15146188

[B41] Richter F. Gabby L. McDowell K. A. Mulligan C. K. De La Rosa K. Sioshansi P. C.. (2017). Effects of decreased dopamine transporter levels on nigrostriatal neurons and paraquat/maneb toxicity in mice. Neurobiol. Aging 51, 54–66. 10.1016/j.neurobiolaging.2016.11.015 28038352 PMC5292275

[B42] Ritz B. R. Manthripragada A. D. Costello S. Lincoln S. J. Farrer M. J. Cockburn M.. (2009). Dopamine transporter genetic variants and pesticides in Parkinson’s disease. Environ. Health Perspect. 117, 964–969. 10.1289/ehp.0800277 19590691 PMC2702414

[B43] Ronzitti G. Bucci G. Emanuele M. Leo D. Sotnikova T. D. Mus L. V.. (2014). Exogenous α-synuclein decreases raft partitioning of Cav2.2 channels inducing dopamine release. J. Neurosci. 34, 10603–10615. 10.1523/JNEUROSCI.0608-14.2014 25100594 PMC6802592

[B44] Senior S. L. Ninkina N. Deacon R. Bannerman D. Buchman V. L. Cragg S. J.. (2008). Increased striatal dopamine release and hyperdopaminergic-like behavior in mice lacking both α-synuclein and γ-synuclein. Eur. J. Neurosci. 27, 947–957. 10.1111/j.1460-9568.2008.06055.x 18333965 PMC3145106

[B45] Siciliano C. A. Saha K. Calipari E. S. Fordahl S. C. Chen R. Khoshbouei H.. (2018). Amphetamine reverses escalated cocaine intake *via* restoration of dopamine transporter conformation. J. Neurosci. 38, 484–497. 10.1523/JNEUROSCI.2604-17.2017 29175958 PMC5761621

[B46] Singleton A. B. Farrer M. Johnson J. Singleton A. Hague S. Kachergus J.. (2003). α-Synuclein locus triplication causes Parkinson’s disease. Science 302:841. 10.1126/science.1090278 14593171

[B47] Sonders M. S. Zhu S. J. Zahniser N. R. Kavanaugh M. P. Amara S. G. (1997). Multiple ionic conductances of the human dopamine transporter: the actions of dopamine and psychostimulants. J. Neurosci. 17, 960–974. 10.1523/JNEUROSCI.17-03-00960.1997 8994051 PMC6573182

[B48] Sulzer D. (2007). Multiple hit hypotheses for dopamine neuron loss in Parkinson’s disease. Trends Neurosci. 30, 244–250. 10.1016/j.tins.2007.03.009 17418429

[B49] Sulzer D. Cragg S. J. Rice M. E. (2016). Striatal dopamine neurotransmission: regulation of release and uptake. Basal Ganglia 6, 123–148. 10.1016/j.baga.2016.02.001 27141430 PMC4850498

[B50] Surmeier D. J. (2018). Determinants of dopaminergic neuron loss in Parkinson’s disease. FEBS J. 285, 3657–3668. 10.1111/febs.14607 30028088 PMC6546423

[B51] Swant J. Goodwin J. S. North A. Ali A. A. Gamble-George J. Chirwa S.. (2011). α-synuclein stimulates a dopamine transporter-dependent chloride current and modulates the activity of the transporter. J. Biol. Chem. 286, 43933–43943. 10.1074/jbc.M111.241232 21990355 PMC3243541

[B52] Threlfell S. Clements M. A. Khodai T. Pienaar I. S. Exley R. Wess J.. (2010). Striatal muscarinic receptors promote activity dependence of dopamine transmission *via* distinct receptor subtypes on cholinergic interneurons in ventral versus dorsal striatum. J. Neurosci. 30, 3398–3408. 10.1523/JNEUROSCI.5620-09.2010 20203199 PMC2866006

[B53] Threlfell S. Lalic T. Platt N. J. Jennings K. A. Deisseroth K. Cragg S. J. (2012). Striatal dopamine release is triggered by synchronized activity in cholinergic interneurons. Neuron 75, 58–64. 10.1016/j.neuron.2012.04.038 22794260

[B54] van de Giessen E. M. de Win M. M. L. Tanck M. W. T. van den Brink W. Baas F. Booij J. (2009). Striatal dopamine transporter availability associated with polymorphisms in the dopamine transporter gene SLC6A3. J. Nucl. Med. 50, 45–52. 10.2967/jnumed.108.053652 19091889

[B55] Vaughan R. A. Foster J. D. (2013). Mechanisms of dopamine transporter regulation in normal and disease states. Trends Pharmacol. Sci. 34, 489–496. 10.1016/j.tips.2013.07.005 23968642 PMC3831354

[B56] Venton B. J. Seipel A. T. Phillips P. E. M. Wetsel W. C. Gitler D. Greengard P.. (2006). Cocaine increases dopamine release by mobilization of a synapsin-dependent reserve pool. J. Neurosci. 26, 3206–3209. 10.1523/JNEUROSCI.4901-04.2006 16554471 PMC6674099

[B57] Wang L. Zhang X. Xu H. Zhou L. Jiao R. Liu W.. (2014). Temporal components of cholinergic terminal to dopaminergic terminal transmission in dorsal striatum slices of mice. J. Physiol. 592, 3559–3576. 10.1113/jphysiol.2014.271825 24973407 PMC4229348

[B58] Wersinger C. Sidhu A. (2003). Attenuation of dopamine transporter activity by α-synuclein. Neurosci. Lett. 340, 189–192. 10.1016/s0304-3940(03)00097-1 12672538

[B59] Zhen J. Reith M. E. A. (2018). Functional properties of dopamine transporter oligomers after copper linking. J. Neurochem. 144, 162–171. 10.1111/jnc.14259 29168892 PMC5760346

