# Supplementary material for: Striatal Dopamine Transporter Function Is Facilitated by Converging Biology of α-Synuclein and Cholesterol
Source: Front Cell Neurosci. 2021 Apr 15;15:658244. doi: 10.3389/fncel.2021.658244 (PMC8081845; doi:10.3389/fncel.2021.658244)
Supplement: Supplementary file 1 [file Data_Sheet_1.pdf]

Supporting information for:

Striatal dopamine transporter function is facilitated by converging biology of α-synuclein and cholesterol

Sarah Threlfell[1,2], Amir Saeid Mohammadi[3], Brent Ryan[1,2], Natalie Connor-Robson[1,2], Nicola J. Platt[1], Rishi Anand[1], Florence Serres[4], Trevor Sharp[4], Nora Bengoa-Vergniory[1,2], Richard Wade-Martins[1,2], Andrew Ewing[5], Stephanie J Cragg[1,2*] and Katherine R Brimblecombe[1,2*]

Contains:

Supplementary Results

Supplementary Figures S1-S5 and Legends

Supplementary Methods

Supplementary References

Supplementary Results

In NAc, cocaine has a greater effect on $[DA]_o$ in *SNCA*-OVX vs *snca*-null mice

It has previously been shown that chronic changes in DA release levels alter DAT levels and function via altered stoichiometry and post-translational modification. To test if the decreased DA release levels in dorsal striatum of OVX mice accounts for the altered DAT response to cocaine we tested the effect of cocaine in NAc, which does not have a DA release deficit (Janezic et al., 2013). Cocaine enhances DA release in NAc of *SNCA*-OVX mice to a greater extent than in *snca*-null mice (**Figure S1**).

DAT function is decreased in DAT-Cre homozygote mice

Dopamine (DA) transporter (DAT) expression levels are decreased in DAT-Cre homozygote ($DAT^{IRESCre}$-homozygous) mice due to disruption of 3' UTR (Bäckman et al., 2006), and illustrated in main manuscript Figure 2D. This decreased DAT level correlated with the decreased functional control of DA release by the DAT: In CPu of DAT-Cre hom mice, cocaine did not significantly increase peak evoked $[DA]_o$ (**Figure S1A**), although it was able to reduce the rate of uptake (**Figure S2A**) and slightly modify the relationship between paired-pulse release and inter-pulse interval (IPI) (**Figure S2B**). These findings extend and are consistent with our previously published findings of altered DAT function in DAT-Cre heterozygote mice (Brimblecombe et al., 2019).

Methyl-β-cyclodextrin does not change the effect of cocaine on dopamine release

We applied water-soluble cholesterol (ws-cholesterol) in methyl-β-cyclodextrin (MBCD) vehicle (1 mM) for experiments illustrated in main manuscript Figure 3A-D. MBCD can deplete membrane cholesterol when used at concentrations higher than used here (Jones et al., 2012). We therefore tested if the effect of cocaine was altered by MBCD (1 mM) compared to drug-free conditions. We found no effect of MBCD on the effect of cocaine on peak evoked $[DA]_o$ for either genotype (**Figure S3**).

Inverse relationship between cholesterol and GalCer with different α-synuclein levels is independent of altered GCase activity

The relationship between different lipid species in both neuronal and glial cells is highly complex (Tracey et al., 2018; Lee et al., 2020), and understanding their individual and interacting roles in health and disease is ongoing. We show that in *SNCA*-OVX mice there is increased GalCer relative to *snca*-null controls in main manuscript Figure 3E,F. It has been shown that in patient samples from the synucleinopathy, multiple systems atrophy (MSA), affected tissue has decreased galactosyceramide (GalCer) level that is inversely proportional to α-synuclein levels (high α-synuclein/low GalCer), thought to be due to demyelination (Don et al., 2014). Additionally, in cultured neurons with high human α-synuclein expression, GalCer levels are

enhanced relative to controls due to compromised lysosomal function (Mazzulli et al., 2016). We therefore tested whether α-synuclein overexpression disrupted lysosomal function leading to an accumulation of GalCer, however we found no difference in enzyme glucocerebrosidase (GCase) activity in the midbrain from *SNCA*-OVX vs *snca*-null controls (**Figure S4**), indicating no impairment of lysosomal function in these mice.

There is no difference in the effect of cocaine on DA release between male and female mice.

To test our assumption that we can combine data from male and female mice we compared the effect of cocaine (5 μM) on peak evoked $[DA]_o$ following a single pulse (1p). We found that there was no difference in the effect of cocaine on DA release between male and female mice, and that the differential effect of cocaine between *SCNA*-OVX and *snca*-null mice is apparent in both sexes (**Figure S5**).

Supplementary Figures

Figure S1: Cocaine increases evoked [DA]$_o$ in NAc to a greater extent in *SNCA*-OVX than *snca*-null mice. Summary of effects of cocaine on mean peak [DA]$_o$ ± SEM evoked by a single stimulus puls, normalised to pre-cocaine condition from NAc of *snca*-null animals (unfilled squares) or *SNCA*-OVX (filled squares). Unpaired t-test: T_{10}=2.65, *P*=0.025.

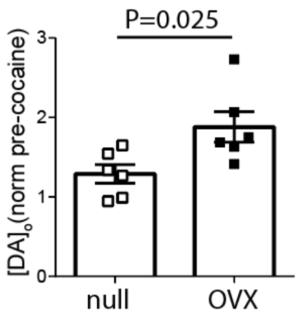

Figure S2: **DAT function is decreased in DAT-Cre hom mice. A)** Mean [DA]$_o$ (normalised to control conditions) ± SEM vs time evoked by single pulses (*arrow*) in CPu in the presence of DHBE, before cocaine (control, black) and in the presence of cocaine (blue, 5 uM). Comparison of normalised peak [DA]$_o$ in cocaine vs hypothetical mean of 1, one-sample t-test, t_2=0.75, *P*=0.53. **B)** Paired-pulse ratios for [DA]$_o$ vs inter-pulse interval (IPI). Two-way ANOVA, drug x IPI interaction, $F_{4,20}$=7.50, *P*=0.0007, Sidak's post-test, **P*<0.05, ***P*<0.01, N=3 recording sites from 3 animals.

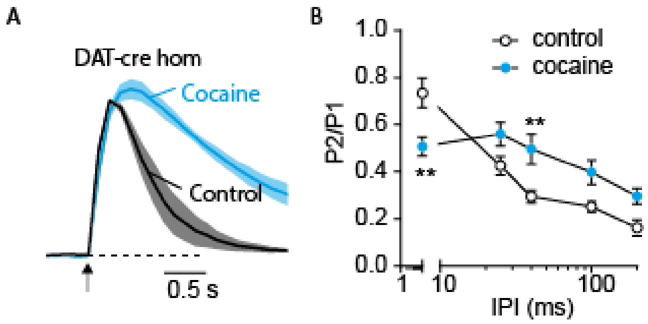

Figure S3: **MBCD does not change the effect of cocaine on DA release.** Summary of effects of cocaine on mean peak [DA]$_o$ ± SEM, normalised to pre-cocaine condition from *snca*-null animals (unfilled circles) or *SNCA*-OVX (filled circles) pre-incubated in either control medium (aCSF, black) or MBCD (grey). Two-way ANOVA: main effect of MBCD, $F_{1,20}$=0.92, P=0.35, N=6 sites from 3 pairs of animals.

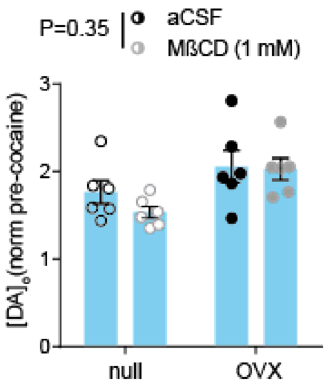

Figure S4: **GCase is not significantly altered in *SNCA*-OVX vs null control.** GCase activity assessed by 4-MUG cleavage in midbrain lysates from *SNCA*-OVX (filled) and *snca*-null control mice (unfilled). T-test, t_{11}=1.713, N=7 *snca*-null and N=6 *SNCA*-OVX.

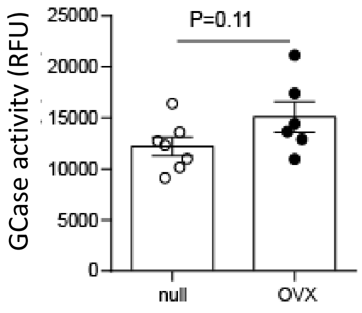

Figure S5: The effect of cocaine on DA release is not different between male and female mice of either *SNCA*-OVX or *snca*-null mice. Summary of effects of cocaine on mean peak $[DA]_o$ ± SEM, normalised to pre-cocaine condition from *snca*-null animals (unfilled circles) or *SNCA*-OVX (filled circles) from female (red) or male (blue) mice. Two-way ANOVA: main effect of sex, $F_{1,30}$=0.10, P=0.84; main effect of genotype, $F_{1,30}$=9.58, P=0.004, N=11 animals for OVX and 6 for nulls.

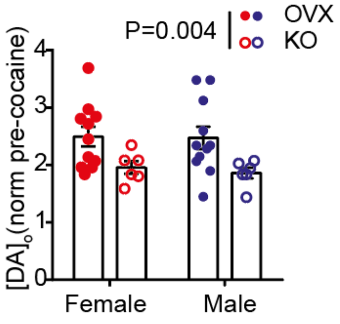

Supplementary Methods

Measurement of GCase activity: GCase activity was assayed as previously described (Fernandes et al., 2016). Briefly, dissected midbrain samples from *snca*-wt and *SNCA*-OVX mice were homogenised and lysed using a Tissue Tearor (Biospec Products, Inc.) in RIPA buffer. Protein concentration was measured using a BCA assay (Sigma) and 5 µg of protein was assayed in citrate-phosphate buffer pH5.4. The cleavage of 4-methylumbelliferyl-β-D-galactopyranoside (4MUG) to fluorescent 4MU was assessed after 60 min incubation in the presence/absence of 1 mM conduritol-β-epoxide (CBE) and read on a plate reader using excitation 360 nm, emission 440 nm (BioTek). GCase activity was calculated by subtracting 4MU fluorescence signal in the presence of CBE from the 4MU signal in the absence of CBE.

Supplementary References

Bäckman, C. M., Malik, N., Zhang, Y., Shan, L., Grinberg, A., Hoffer, B. J., et al. (2006). Characterization of a mouse strain expressing Cre recombinase from the 3' untranslated region of the dopamine transporter locus. *Genesis* 44, 383–90. doi:10.1002/dvg.20228.

Brimblecombe, K. R., Vietti-Michelina, S., Platt, N. J., Kastli, R., Hnieno, A., Gracie, C. J., et al. (2019). Calbindin-D28K Limits Dopamine Release in Ventral but Not Dorsal Striatum by Regulating Ca 2+ Availability and Dopamine Transporter Function. *ACS Chem. Neurosci.* 10, 3419–3426. doi:10.1021/acschemneuro.9b00325.

Don, A. S., Hsiao, J. H. T., Bleasel, J. M., Couttas, T. A., Halliday, G. M., and Kim, W. S. (2014). Altered lipid levels provide evidence for myelin dysfunction in multiple system atrophy. *Acta Neuropathol. Commun.* 2, 150. doi:10.1186/s40478-014-0150-6.

Fernandes, H. J. R., Hartfield, E. M., Christian, H. C., Emmanoulidou, E., Zheng, Y., Booth, H., et al. (2016). ER Stress and Autophagic Perturbations Lead to Elevated Extracellular α-Synuclein in GBA-N370S Parkinson's iPSC-Derived Dopamine Neurons. *Stem Cell Reports* 6, 342–356. doi:10.1016/j.stemcr.2016.01.013.

Jones, K. T., Zhen, J., and Reith, M. E. A. (2012). Importance of cholesterol in dopamine transporter function. *J. Neurochem.* 123, 700–15. doi:10.1111/jnc.12007.

Lee, J. A., Hall, B., Allsop, J., Alqarni, R., and Allen, S. P. (2020). Lipid metabolism in astrocytic structure and function. *Semin. Cell Dev. Biol.* doi:10.1016/j.semcdb.2020.07.017.

Mazzulli, J. R., Zunke, F., Isacson, O., Studer, L., and Krainc, D. (2016). α-Synuclein-induced lysosomal dysfunction occurs through disruptions in protein trafficking in human midbrain synucleinopathy models. *Proc. Natl. Acad. Sci. U. S. A.* 113, 1931–1936. doi:10.1073/pnas.1520335113.

Tracey, T. J., Steyn, F. J., Wolvetang, E. J., and Ngo, S. T. (2018). Neuronal lipid metabolism: Multiple pathways driving functional outcomes in health and disease. *Front. Mol. Neurosci.* 11.10. doi:10.3389/fnmol.2018.00010.
